# 2,4-Diethyl­thioxanthen-9-one

**DOI:** 10.1107/S1600536809006308

**Published:** 2009-02-28

**Authors:** Ge Liu

**Affiliations:** a Chifeng University, Chifeng 024000, People’s Republic of China

## Abstract

The asymmetric unit of the title compound, C_17_H_16_OS, contains two crystallographically independent mol­ecules, one of which is nearly planar, the outer rings making dihedral angles of 1.51 (3) and 1.09 (3)° with the central ring. In the crystal structure, weak inter­molecular C—H⋯O hydrogen bonds link mol­ecules into chains parallel to the *a* axis. π–π Contacts between the thioxanthone rings [centroid–centroid distances = 3.798 (3) and 3.781 (3) Å] may further stabilize the structure.

## Related literature

For general background, see: Fouassier *et al.* (1995 ▶); Roffey (1997 ▶). For a related structure, see: Bearson *et al.* (1996 ▶). For bond-length data, see: Allen *et al.* (1987 ▶).
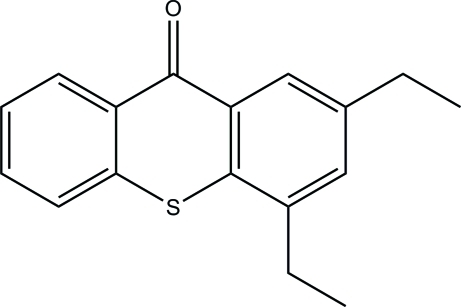


## Experimental

### 

#### Crystal data

C_17_H_16_OS
*M* *_r_* = 268.37
Triclinic, 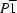

*a* = 9.5403 (19) Å
*b* = 11.083 (2) Å
*c* = 13.807 (3) Å
α = 77.19 (3)°
β = 87.72 (3)°
γ = 79.35 (3)°
*V* = 1399.0 (5) Å^3^
*Z* = 4
Mo *K*α radiation
μ = 0.22 mm^−1^
*T* = 294 K
0.15 × 0.12 × 0.10 mm

#### Data collection

Rigaku R-AXIS RAPID-S diffractometer
Absorption correction: none
12071 measured reflections
4920 independent reflections
3346 reflections with *I* > 2σ(*I*)
*R*_int_ = 0.041

#### Refinement

*R*[*F* ^2^ > 2σ(*F* ^2^)] = 0.064
*wR*(*F* ^2^) = 0.138
*S* = 1.07
4920 reflections
344 parameters
H-atom parameters constrained
Δρ_max_ = 0.30 e Å^−3^
Δρ_min_ = −0.27 e Å^−3^

### 

Data collection: *RAPID-AUTO* (Rigaku, 1998 ▶); cell refinement: *RAPID-AUTO*; data reduction: *CrystalStructure* (Rigaku/MSC, 2002 ▶); program(s) used to solve structure: *SHELXS97* (Sheldrick, 2008 ▶); program(s) used to refine structure: *SHELXL97* (Sheldrick, 2008 ▶); molecular graphics: *SHELXTL* (Sheldrick, 2008 ▶) and *ORTEP-3 for Windows* (Farrugia, 1997 ▶); software used to prepare material for publication: *SHELXTL* and *PLATON* (Spek, 2009 ▶).

## Supplementary Material

Crystal structure: contains datablocks I, global. DOI: 10.1107/S1600536809006308/hk2627sup1.cif

Structure factors: contains datablocks I. DOI: 10.1107/S1600536809006308/hk2627Isup2.hkl

Additional supplementary materials:  crystallographic information; 3D view; checkCIF report

## Figures and Tables

**Table 1:** Hydrogen-bond geometry (Å, °)

*D*—H⋯*A*	*D*—H	H⋯*A*	*D*⋯*A*	*D*—H⋯*A*
C14—H14*A*⋯O2^i^	0.93	2.54	3.334 (3)	143
C32—H32*A*⋯O1^ii^	0.93	2.61	3.466 (3)	154

Symmetry codes: (i) 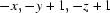; (ii) 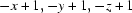.

**Fig. 1.**
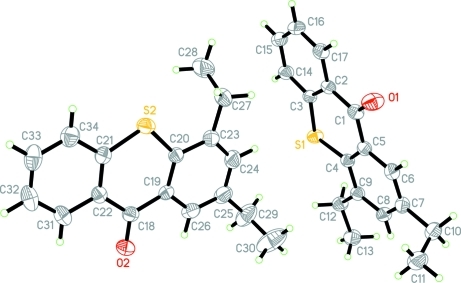
The molecular structure of the title molecule, with the atom-numbering scheme. Displacement ellipsoids are drawn at the 30% probability level.

**Fig. 2.**
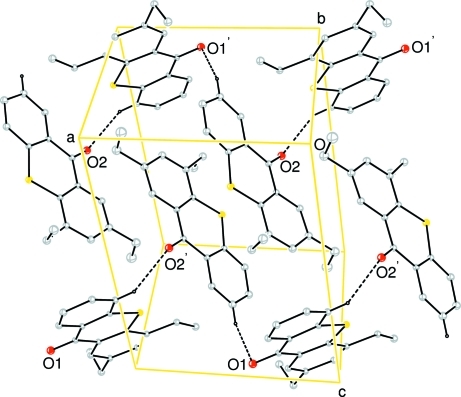
A partial packing diagram of the title compound [symmetry code ('): -x, -y, -z]. Hydrogen bonds are shown as dashed lines.

## supplementary crystallographic information

### Comment

Thioxanthone derivatives are good photoinitiators with excellent capabilities in UV-curing materials. They have been widely used in UV-curing applications because they absorb at a longer UV wavelength and have a faster photocuring speed than other photoinitiators. They have been introduced in processes such as printing inks, surface coatings, microelectronics and photoresists (Fouassier *et al.*, 1995; Roffey, 1997). We report herein the crystal structure of the title compound.

The asymmetric unit of the title compound contains two crystallographically independent molecules (Fig. 1), in which the bond lengths (Allen *et al.*, 1987) and angles are generally within normal ranges. Rings A (S1/C1-C5), B (C4-C9), C (C2/C3/C14-C17) and D (S2/C18-C22), E (C19/C20/C23-C26), F (C21/C22/C31-C34) are, of course, planar, and they are oriented at dihedral angles of A/B = 1.51 (3), A/C = 1.09 (3), B/C = 2.54 (3) ° and D/E = 2.95 (3), D/F = 2.85 (3), E/F = 5.75 (3) °. So, rings A, B and C are nearly coplanar.

In the crystal structure, weak intermolecular C-H···O hydrogen bonds (Table 1) link the molecules into chains parallel to the a-axis (Fig. 2), in which they may be effective in the stabilization of the structure. The π-π contacts between the thioxanthone rings, Cg1—Cg2^i^ and Cg4—Cg4^ii^ [symmetry codes: (i) -x, -y, 2 - z; (ii) 1 - x, 1 - y, 1 - z, where Cg1, Cg2 and Cg4 are centroids of the rings A (S1/C1-C5), B (C4-C9) and D (S2/C18-C22), respectively] may further stabilize the structure, with centroid-centroid distances of 3.798 (3) and 3.781 (3) Å, respectively.

### Experimental

The title compound was prepared by the literature method (Bearson *et al.*, 1996). Crystals suitable for X-ray analysis were obtained by dissolving the title compound (80.5 mg, 0.3 mmol) in ethanol (10 ml) and evaporating ethanol slowly at room temperature for about 10 d.

### Refinement

H atoms were positioned geometrically, with C-H = 0.93, 0.97 and 0.96 Å for aromatic, methylene and methyl H, respectively, and constrained to ride on their parent atoms, with U_iso_(H) = xU_eq_(C), where x = 1.5 for methyl H and x = 1.2 for all other H atoms.

### Crystal data

**Table:**

C_17_H_16_OS	*Z* = 4
*M**_r_* = 268.37	*F*(000) = 568
Triclinic, *P*1	*D*_x_ = 1.274 Mg m^−^^3^
Hall symbol: -P 1	Mo *K*α radiation, λ = 0.71073 Å
*a* = 9.5403 (19) Å	Cell parameters from 10591 reflections
*b* = 11.083 (2) Å	θ = 3.0–27.7°
*c* = 13.807 (3) Å	µ = 0.22 mm^−^^1^
α = 77.19 (3)°	*T* = 294 K
β = 87.72 (3)°	Block, yellow
γ = 79.35 (3)°	0.15 × 0.12 × 0.10 mm
*V* = 1399.0 (5) Å^3^	

### Data collection

**Table:**

Rigaku R-AXIS RAPID-S diffractometer	3346 reflections with *I* > 2σ(*I*)
Radiation source: fine-focus sealed tube	*R*_int_ = 0.041
graphite	θ_max_ = 25.0°, θ_min_ = 3.0°
ω scans	*h* = −11→11
12071 measured reflections	*k* = −13→13
4920 independent reflections	*l* = −16→16

### Refinement

**Table:**

Refinement on *F*^2^	Primary atom site location: structure-invariant direct methods
Least-squares matrix: full	Secondary atom site location: difference Fourier map
*R*[*F*^2^ > 2σ(*F*^2^)] = 0.064	Hydrogen site location: inferred from neighbouring sites
*wR*(*F*^2^) = 0.138	H-atom parameters constrained
*S* = 1.07	*w* = 1/[σ^2^(*F*_o_^2^) + (0.0457*P*)^2^ + 0.6633*P*] where *P* = (*F*_o_^2^ + 2*F*_c_^2^)/3
4920 reflections	(Δ/σ)_max_ < 0.001
344 parameters	Δρ_max_ = 0.30 e Å^−^^3^
0 restraints	Δρ_min_ = −0.27 e Å^−^^3^

### Special details

**Table:**

Geometry. All e.s.d.'s (except the e.s.d. in the dihedral angle between two l.s. planes) are estimated using the full covariance matrix. The cell e.s.d.'s are taken into account individually in the estimation of e.s.d.'s in distances, angles and torsion angles; correlations between e.s.d.'s in cell parameters are only used when they are defined by crystal symmetry. An approximate (isotropic) treatment of cell e.s.d.'s is used for estimating e.s.d.'s involving l.s. planes.
Refinement. Refinement of *F*^2^ against ALL reflections. The weighted *R*-factor *wR* and goodness of fit *S* are based on *F*^2^, conventional *R*-factors *R* are based on *F*, with *F* set to zero for negative *F*^2^. The threshold expression of *F*^2^ > σ(*F*^2^) is used only for calculating *R*-factors(gt) *etc*. and is not relevant to the choice of reflections for refinement. *R*-factors based on *F*^2^ are statistically about twice as large as those based on *F*, and *R*- factors based on ALL data will be even larger.

### Fractional atomic coordinates and isotropic or equivalent isotropic displacement parameters (Å^2^)

**Table:**

	*x*	*y*	*z*	*U*_iso_*/*U*_eq_	
S1	−0.05043 (8)	0.12220 (8)	0.80789 (6)	0.0520 (2)	
S2	0.44522 (9)	0.39943 (8)	0.38211 (7)	0.0609 (3)	
O1	0.3940 (2)	−0.0576 (2)	0.89662 (19)	0.0776 (7)	
O2	0.2369 (3)	0.7698 (2)	0.43252 (18)	0.0775 (7)	
C1	0.2704 (3)	−0.0079 (3)	0.8721 (2)	0.0514 (8)	
C2	0.2069 (3)	−0.0339 (3)	0.7856 (2)	0.0453 (7)	
C3	0.0682 (3)	0.0190 (3)	0.7522 (2)	0.0453 (7)	
C4	0.0448 (3)	0.1358 (3)	0.9095 (2)	0.0443 (7)	
C5	0.1864 (3)	0.0751 (3)	0.9311 (2)	0.0465 (7)	
C6	0.2526 (3)	0.0939 (3)	1.0140 (2)	0.0550 (8)	
H6A	0.3469	0.0553	1.0280	0.066*	
C7	0.1827 (4)	0.1672 (3)	1.0750 (2)	0.0562 (8)	
C8	0.0407 (4)	0.2253 (3)	1.0513 (2)	0.0578 (8)	
H8A	−0.0079	0.2750	1.0925	0.069*	
C9	−0.0300 (3)	0.2125 (3)	0.9707 (2)	0.0490 (8)	
C10	0.2553 (4)	0.1865 (3)	1.1643 (3)	0.0711 (10)	
H10A	0.1845	0.1996	1.2151	0.085*	
H10B	0.3237	0.1113	1.1912	0.085*	
C11	0.3296 (4)	0.2955 (4)	1.1394 (3)	0.0839 (12)	
H11A	0.3730	0.3049	1.1982	0.126*	
H11B	0.2622	0.3704	1.1133	0.126*	
H11C	0.4018	0.2818	1.0906	0.126*	
C12	−0.1830 (3)	0.2763 (3)	0.9459 (2)	0.0595 (9)	
H12A	−0.1851	0.3292	0.8796	0.071*	
H12B	−0.2388	0.2119	0.9443	0.071*	
C13	−0.2552 (4)	0.3561 (3)	1.0161 (3)	0.0764 (11)	
H13A	−0.3511	0.3920	0.9940	0.115*	
H13B	−0.2033	0.4223	1.0169	0.115*	
H13C	−0.2569	0.3046	1.0818	0.115*	
C14	0.0149 (3)	−0.0109 (3)	0.6691 (2)	0.0561 (8)	
H14A	−0.0777	0.0243	0.6473	0.067*	
C15	0.0988 (4)	−0.0917 (3)	0.6202 (3)	0.0643 (9)	
H15A	0.0629	−0.1115	0.5652	0.077*	
C16	0.2371 (4)	−0.1445 (3)	0.6517 (3)	0.0663 (10)	
H16A	0.2941	−0.1992	0.6177	0.080*	
C17	0.2892 (3)	−0.1158 (3)	0.7328 (2)	0.0595 (9)	
H17A	0.3821	−0.1517	0.7536	0.071*	
C18	0.2905 (3)	0.6672 (3)	0.4167 (2)	0.0542 (8)	
C19	0.2565 (3)	0.5507 (3)	0.4814 (2)	0.0499 (8)	
C20	0.3206 (3)	0.4300 (3)	0.4732 (2)	0.0501 (8)	
C21	0.4573 (3)	0.5467 (3)	0.3105 (2)	0.0522 (8)	
C22	0.3880 (3)	0.6599 (3)	0.3316 (2)	0.0500 (8)	
C23	0.2871 (4)	0.3240 (3)	0.5413 (3)	0.0633 (9)	
C24	0.1856 (4)	0.3452 (4)	0.6124 (3)	0.0777 (11)	
H24A	0.1627	0.2758	0.6574	0.093*	
C25	0.1153 (4)	0.4639 (4)	0.6209 (3)	0.0777 (11)	
C26	0.1542 (4)	0.5643 (3)	0.5557 (3)	0.0664 (10)	
H26A	0.1109	0.6450	0.5608	0.080*	
C27	0.3561 (5)	0.1909 (3)	0.5352 (3)	0.0816 (12)	
H27A	0.4561	0.1890	0.5187	0.098*	
H27B	0.3505	0.1354	0.5997	0.098*	
C28	0.2864 (5)	0.1424 (4)	0.4588 (3)	0.1110 (16)	
H28A	0.3345	0.0587	0.4574	0.166*	
H28B	0.2925	0.1964	0.3946	0.166*	
H28C	0.1881	0.1414	0.4759	0.166*	
C29	−0.0003 (5)	0.4844 (5)	0.6996 (3)	0.1119 (17)	
H29A	−0.0737	0.5547	0.6704	0.134*	
H29B	−0.0443	0.4103	0.7174	0.134*	
C30	0.0492 (6)	0.5081 (6)	0.7860 (4)	0.149 (2)	
H30A	−0.0290	0.5206	0.8308	0.223*	
H30B	0.0915	0.5822	0.7696	0.223*	
H30C	0.1193	0.4376	0.8171	0.223*	
C31	0.4111 (4)	0.7717 (3)	0.2682 (3)	0.0639 (9)	
H31A	0.3668	0.8483	0.2816	0.077*	
C32	0.4975 (4)	0.7706 (4)	0.1867 (3)	0.0789 (12)	
H32A	0.5112	0.8460	0.1452	0.095*	
C33	0.5642 (4)	0.6576 (5)	0.1661 (3)	0.0829 (12)	
H33A	0.6225	0.6569	0.1105	0.099*	
C34	0.5451 (4)	0.5471 (4)	0.2270 (3)	0.0709 (10)	
H34A	0.5908	0.4713	0.2129	0.085*	

### Atomic displacement parameters (Å^2^)

**Table:**

	*U*^11^	*U*^22^	*U*^33^	*U*^12^	*U*^13^	*U*^23^
S1	0.0420 (4)	0.0550 (5)	0.0615 (5)	−0.0026 (4)	−0.0015 (4)	−0.0228 (4)
S2	0.0662 (6)	0.0485 (5)	0.0674 (6)	−0.0057 (4)	0.0047 (4)	−0.0166 (4)
O1	0.0514 (14)	0.0868 (18)	0.0989 (19)	0.0108 (13)	−0.0142 (13)	−0.0459 (15)
O2	0.106 (2)	0.0464 (14)	0.0747 (17)	0.0024 (13)	0.0078 (14)	−0.0170 (12)
C1	0.0417 (18)	0.0479 (19)	0.063 (2)	−0.0035 (15)	−0.0013 (15)	−0.0117 (16)
C2	0.0421 (17)	0.0393 (17)	0.053 (2)	−0.0049 (14)	0.0026 (14)	−0.0094 (14)
C3	0.0500 (18)	0.0387 (17)	0.0473 (19)	−0.0078 (14)	0.0048 (14)	−0.0107 (13)
C4	0.0464 (17)	0.0392 (17)	0.0474 (19)	−0.0108 (14)	0.0051 (14)	−0.0084 (13)
C5	0.0447 (18)	0.0444 (17)	0.0499 (19)	−0.0088 (14)	0.0008 (14)	−0.0088 (14)
C6	0.0531 (19)	0.054 (2)	0.056 (2)	−0.0091 (16)	−0.0034 (16)	−0.0063 (16)
C7	0.062 (2)	0.057 (2)	0.049 (2)	−0.0121 (17)	−0.0038 (16)	−0.0084 (16)
C8	0.068 (2)	0.054 (2)	0.052 (2)	−0.0087 (17)	0.0076 (17)	−0.0167 (16)
C9	0.0491 (18)	0.0455 (18)	0.051 (2)	−0.0063 (15)	0.0024 (15)	−0.0105 (14)
C10	0.080 (3)	0.076 (3)	0.058 (2)	−0.008 (2)	−0.0083 (19)	−0.0192 (19)
C11	0.091 (3)	0.085 (3)	0.083 (3)	−0.017 (2)	−0.014 (2)	−0.031 (2)
C12	0.057 (2)	0.058 (2)	0.062 (2)	−0.0022 (16)	0.0087 (16)	−0.0204 (17)
C13	0.073 (2)	0.073 (3)	0.078 (3)	0.010 (2)	0.0063 (19)	−0.025 (2)
C14	0.055 (2)	0.053 (2)	0.059 (2)	−0.0010 (16)	−0.0046 (16)	−0.0164 (16)
C15	0.074 (2)	0.059 (2)	0.060 (2)	−0.0007 (19)	−0.0069 (18)	−0.0224 (18)
C16	0.074 (2)	0.057 (2)	0.065 (2)	0.0071 (19)	0.0036 (19)	−0.0253 (18)
C17	0.054 (2)	0.056 (2)	0.063 (2)	0.0049 (16)	−0.0005 (16)	−0.0121 (17)
C18	0.059 (2)	0.050 (2)	0.054 (2)	−0.0042 (17)	−0.0154 (16)	−0.0142 (16)
C19	0.059 (2)	0.051 (2)	0.0440 (19)	−0.0134 (16)	−0.0034 (15)	−0.0153 (15)
C20	0.0575 (19)	0.0508 (19)	0.0447 (19)	−0.0147 (16)	−0.0066 (14)	−0.0108 (15)
C21	0.0472 (18)	0.061 (2)	0.049 (2)	−0.0108 (16)	−0.0042 (15)	−0.0114 (15)
C22	0.0477 (18)	0.052 (2)	0.050 (2)	−0.0111 (15)	−0.0106 (15)	−0.0061 (15)
C23	0.081 (2)	0.058 (2)	0.056 (2)	−0.0234 (19)	−0.0054 (18)	−0.0120 (17)
C24	0.109 (3)	0.076 (3)	0.058 (2)	−0.047 (2)	0.007 (2)	−0.010 (2)
C25	0.098 (3)	0.087 (3)	0.064 (3)	−0.044 (3)	0.016 (2)	−0.029 (2)
C26	0.072 (2)	0.070 (2)	0.066 (2)	−0.0183 (19)	0.0078 (19)	−0.031 (2)
C27	0.117 (3)	0.057 (2)	0.072 (3)	−0.028 (2)	−0.004 (2)	−0.0056 (19)
C28	0.159 (5)	0.072 (3)	0.113 (4)	−0.045 (3)	−0.016 (3)	−0.021 (3)
C29	0.126 (4)	0.162 (5)	0.076 (3)	−0.079 (4)	0.037 (3)	−0.047 (3)
C30	0.117 (4)	0.236 (7)	0.140 (5)	−0.067 (4)	0.053 (4)	−0.120 (5)
C31	0.059 (2)	0.055 (2)	0.072 (3)	−0.0135 (17)	−0.0111 (18)	0.0026 (18)
C32	0.063 (2)	0.082 (3)	0.079 (3)	−0.024 (2)	−0.005 (2)	0.017 (2)
C33	0.062 (2)	0.112 (4)	0.065 (3)	−0.017 (2)	0.0095 (19)	0.001 (2)
C34	0.062 (2)	0.081 (3)	0.068 (3)	−0.011 (2)	0.0078 (19)	−0.015 (2)

### Geometric parameters (Å, °)

**Table:**

C1—O1	1.232 (3)	C18—O2	1.220 (3)
C1—C2	1.467 (4)	C18—C22	1.477 (4)
C1—C5	1.470 (4)	C18—C19	1.479 (4)
C2—C3	1.396 (4)	C19—C20	1.392 (4)
C2—C17	1.398 (4)	C19—C26	1.401 (4)
C3—C14	1.402 (4)	C20—C23	1.411 (4)
C3—S1	1.739 (3)	C20—S2	1.741 (3)
C4—C5	1.405 (4)	C21—C22	1.392 (4)
C4—C9	1.411 (4)	C21—C34	1.398 (4)
C4—S1	1.750 (3)	C21—S2	1.732 (3)
C5—C6	1.400 (4)	C22—C31	1.398 (4)
C6—C7	1.368 (4)	C23—C24	1.379 (5)
C6—H6A	0.9300	C23—C27	1.520 (5)
C7—C8	1.407 (4)	C24—C25	1.390 (5)
C7—C10	1.511 (4)	C24—H24A	0.9300
C8—C9	1.371 (4)	C25—C26	1.367 (5)
C8—H8A	0.9300	C25—C29	1.540 (5)
C9—C12	1.516 (4)	C26—H26A	0.9300
C10—C11	1.480 (5)	C27—C28	1.510 (5)
C10—H10A	0.9700	C27—H27A	0.9700
C10—H10B	0.9700	C27—H27B	0.9700
C11—H11A	0.9600	C28—H28A	0.9600
C11—H11B	0.9600	C28—H28B	0.9600
C11—H11C	0.9600	C28—H28C	0.9600
C12—C13	1.516 (4)	C29—C30	1.395 (6)
C12—H12A	0.9700	C29—H29A	0.9700
C12—H12B	0.9700	C29—H29B	0.9700
C13—H13A	0.9600	C30—H30A	0.9600
C13—H13B	0.9600	C30—H30B	0.9600
C13—H13C	0.9600	C30—H30C	0.9600
C14—C15	1.363 (4)	C31—C32	1.370 (5)
C14—H14A	0.9300	C31—H31A	0.9300
C15—C16	1.386 (5)	C32—C33	1.379 (5)
C15—H15A	0.9300	C32—H32A	0.9300
C16—C17	1.366 (4)	C33—C34	1.362 (5)
C16—H16A	0.9300	C33—H33A	0.9300
C17—H17A	0.9300	C34—H34A	0.9300
			
O1—C1—C2	119.9 (3)	O2—C18—C19	120.3 (3)
O1—C1—C5	119.7 (3)	C22—C18—C19	120.1 (3)
C2—C1—C5	120.4 (3)	C20—C19—C26	118.7 (3)
C3—C2—C17	117.7 (3)	C20—C19—C18	124.0 (3)
C3—C2—C1	123.7 (3)	C26—C19—C18	117.3 (3)
C17—C2—C1	118.7 (3)	C19—C20—C23	120.3 (3)
C2—C3—C14	120.3 (3)	C19—C20—S2	123.5 (2)
C2—C3—S1	124.5 (2)	C23—C20—S2	116.2 (2)
C14—C3—S1	115.1 (2)	C22—C21—C34	119.9 (3)
C5—C4—C9	120.8 (3)	C22—C21—S2	124.6 (2)
C5—C4—S1	123.5 (2)	C34—C21—S2	115.5 (3)
C9—C4—S1	115.7 (2)	C21—C22—C31	118.1 (3)
C6—C5—C4	118.6 (3)	C21—C22—C18	123.1 (3)
C6—C5—C1	117.5 (3)	C31—C22—C18	118.8 (3)
C4—C5—C1	124.0 (3)	C24—C23—C20	117.5 (3)
C7—C6—C5	122.1 (3)	C24—C23—C27	120.7 (3)
C7—C6—H6A	119.0	C20—C23—C27	121.8 (3)
C5—C6—H6A	119.0	C23—C24—C25	124.0 (4)
C6—C7—C8	117.6 (3)	C23—C24—H24A	118.0
C6—C7—C10	121.6 (3)	C25—C24—H24A	118.0
C8—C7—C10	120.8 (3)	C26—C25—C24	116.7 (4)
C9—C8—C7	123.4 (3)	C26—C25—C29	120.5 (4)
C9—C8—H8A	118.3	C24—C25—C29	122.8 (4)
C7—C8—H8A	118.3	C25—C26—C19	122.8 (3)
C8—C9—C4	117.6 (3)	C25—C26—H26A	118.6
C8—C9—C12	122.8 (3)	C19—C26—H26A	118.6
C4—C9—C12	119.7 (3)	C28—C27—C23	112.8 (3)
C11—C10—C7	112.3 (3)	C28—C27—H27A	109.0
C11—C10—H10A	109.1	C23—C27—H27A	109.0
C7—C10—H10A	109.1	C28—C27—H27B	109.0
C11—C10—H10B	109.1	C23—C27—H27B	109.0
C7—C10—H10B	109.1	H27A—C27—H27B	107.8
H10A—C10—H10B	107.9	C27—C28—H28A	109.5
C10—C11—H11A	109.5	C27—C28—H28B	109.5
C10—C11—H11B	109.5	H28A—C28—H28B	109.5
H11A—C11—H11B	109.5	C27—C28—H28C	109.5
C10—C11—H11C	109.5	H28A—C28—H28C	109.5
H11A—C11—H11C	109.5	H28B—C28—H28C	109.5
H11B—C11—H11C	109.5	C30—C29—C25	114.5 (4)
C13—C12—C9	116.0 (3)	C30—C29—H29A	108.6
C13—C12—H12A	108.3	C25—C29—H29A	108.6
C9—C12—H12A	108.3	C30—C29—H29B	108.6
C13—C12—H12B	108.3	C25—C29—H29B	108.6
C9—C12—H12B	108.3	H29A—C29—H29B	107.6
H12A—C12—H12B	107.4	C29—C30—H30A	109.5
C12—C13—H13A	109.5	C29—C30—H30B	109.5
C12—C13—H13B	109.5	H30A—C30—H30B	109.5
H13A—C13—H13B	109.5	C29—C30—H30C	109.5
C12—C13—H13C	109.5	H30A—C30—H30C	109.5
H13A—C13—H13C	109.5	H30B—C30—H30C	109.5
H13B—C13—H13C	109.5	C32—C31—C22	121.3 (4)
C15—C14—C3	119.9 (3)	C32—C31—H31A	119.3
C15—C14—H14A	120.0	C22—C31—H31A	119.3
C3—C14—H14A	120.0	C31—C32—C33	119.9 (4)
C14—C15—C16	120.6 (3)	C31—C32—H32A	120.0
C14—C15—H15A	119.7	C33—C32—H32A	120.0
C16—C15—H15A	119.7	C34—C33—C32	120.2 (4)
C17—C16—C15	119.5 (3)	C34—C33—H33A	119.9
C17—C16—H16A	120.2	C32—C33—H33A	119.9
C15—C16—H16A	120.2	C33—C34—C21	120.6 (4)
C16—C17—C2	121.9 (3)	C33—C34—H34A	119.7
C16—C17—H17A	119.1	C21—C34—H34A	119.7
C2—C17—H17A	119.1	C3—S1—C4	103.87 (14)
O2—C18—C22	119.5 (3)	C21—S2—C20	104.32 (15)
			
O1—C1—C2—C3	−179.5 (3)	C26—C19—C20—C23	−2.6 (4)
C5—C1—C2—C3	1.8 (4)	C18—C19—C20—C23	176.7 (3)
O1—C1—C2—C17	0.2 (5)	C26—C19—C20—S2	178.0 (2)
C5—C1—C2—C17	−178.5 (3)	C18—C19—C20—S2	−2.6 (4)
C17—C2—C3—C14	0.5 (4)	C34—C21—C22—C31	−1.0 (4)
C1—C2—C3—C14	−179.7 (3)	S2—C21—C22—C31	178.3 (2)
C17—C2—C3—S1	179.8 (2)	C34—C21—C22—C18	178.2 (3)
C1—C2—C3—S1	−0.5 (4)	S2—C21—C22—C18	−2.4 (4)
C9—C4—C5—C6	−1.0 (4)	O2—C18—C22—C21	177.7 (3)
S1—C4—C5—C6	179.4 (2)	C19—C18—C22—C21	−3.3 (4)
C9—C4—C5—C1	178.8 (3)	O2—C18—C22—C31	−3.0 (4)
S1—C4—C5—C1	−0.8 (4)	C19—C18—C22—C31	176.0 (3)
O1—C1—C5—C6	−0.1 (4)	C19—C20—C23—C24	2.5 (5)
C2—C1—C5—C6	178.7 (3)	S2—C20—C23—C24	−178.1 (3)
O1—C1—C5—C4	−179.8 (3)	C19—C20—C23—C27	−179.4 (3)
C2—C1—C5—C4	−1.1 (4)	S2—C20—C23—C27	−0.1 (4)
C4—C5—C6—C7	1.2 (5)	C20—C23—C24—C25	−0.2 (6)
C1—C5—C6—C7	−178.6 (3)	C27—C23—C24—C25	−178.3 (4)
C5—C6—C7—C8	−0.6 (5)	C23—C24—C25—C26	−1.9 (6)
C5—C6—C7—C10	179.9 (3)	C23—C24—C25—C29	178.5 (4)
C6—C7—C8—C9	−0.3 (5)	C24—C25—C26—C19	1.8 (6)
C10—C7—C8—C9	179.1 (3)	C29—C25—C26—C19	−178.6 (3)
C7—C8—C9—C4	0.5 (5)	C20—C19—C26—C25	0.4 (5)
C7—C8—C9—C12	−179.7 (3)	C18—C19—C26—C25	−179.0 (3)
C5—C4—C9—C8	0.1 (4)	C24—C23—C27—C28	96.8 (4)
S1—C4—C9—C8	179.8 (2)	C20—C23—C27—C28	−81.2 (4)
C5—C4—C9—C12	−179.7 (3)	C26—C25—C29—C30	−84.2 (6)
S1—C4—C9—C12	0.0 (4)	C24—C25—C29—C30	95.4 (6)
C6—C7—C10—C11	89.0 (4)	C21—C22—C31—C32	0.9 (5)
C8—C7—C10—C11	−90.4 (4)	C18—C22—C31—C32	−178.3 (3)
C8—C9—C12—C13	−0.1 (5)	C22—C31—C32—C33	−0.3 (5)
C4—C9—C12—C13	179.7 (3)	C31—C32—C33—C34	−0.4 (6)
C2—C3—C14—C15	−0.2 (5)	C32—C33—C34—C21	0.3 (6)
S1—C3—C14—C15	−179.6 (3)	C22—C21—C34—C33	0.4 (5)
C3—C14—C15—C16	−0.2 (5)	S2—C21—C34—C33	−179.0 (3)
C14—C15—C16—C17	0.4 (5)	C2—C3—S1—C4	−1.2 (3)
C15—C16—C17—C2	−0.1 (5)	C14—C3—S1—C4	178.2 (2)
C3—C2—C17—C16	−0.3 (5)	C5—C4—S1—C3	1.8 (3)
C1—C2—C17—C16	179.9 (3)	C9—C4—S1—C3	−177.9 (2)
O2—C18—C19—C20	−175.1 (3)	C22—C21—S2—C20	4.8 (3)
C22—C18—C19—C20	5.9 (4)	C34—C21—S2—C20	−175.8 (2)
O2—C18—C19—C26	4.3 (4)	C19—C20—S2—C21	−2.3 (3)
C22—C18—C19—C26	−174.8 (3)	C23—C20—S2—C21	178.3 (2)

### Hydrogen-bond geometry (Å, °)

**Table:**

*D*—H···*A*	*D*—H	H···*A*	*D*···*A*	*D*—H···*A*
C14—H14A···O2^i^	0.93	2.54	3.334 (3)	143
C32—H32A···O1^ii^	0.93	2.61	3.466 (3)	154

Symmetry codes: (i) −*x*, −*y*+1, −*z*+1; (ii) −*x*+1, −*y*+1, −*z*+1.
